# INO80 function is required for mouse mammary gland development, but mutation alone may be insufficient for breast cancer

**DOI:** 10.3389/fcell.2023.1253274

**Published:** 2023-11-01

**Authors:** Nguyen Xuan Thang, Dong Wook Han, Chanhyeok Park, Hyeonji Lee, Hyeonwoo La, Seonho Yoo, Heeji Lee, Sang Jun Uhm, Hyuk Song, Jeong Tae Do, Kyoung Sik Park, Youngsok Choi, Kwonho Hong

**Affiliations:** ^1^ Department of Stem Cell and Regenerative Biotechnology, Institute of Advanced Regenerative Science, Konkuk University, Seoul, Republic of Korea; ^2^ Guangdong Provincial Key Laboratory of Large Animal Models for Biomedicine, Wuyi University, Jiangmen, China; ^3^ Department of Animal Science, Sangji University, Wonju, Republic of Korea; ^4^ Department of Surgery, School of Medicine, Konkuk University, Seoul, Republic of Korea

**Keywords:** mammary gland development, breast cancer, INO80, transcriptional regulation, estrogen

## Abstract

The aberrant function of ATP-dependent chromatin remodeler INO80 has been implicated in multiple types of cancers by altering chromatin architecture and gene expression; however, the underlying mechanism of the functional involvement of INO80 mutation in cancer etiology, especially in breast cancer, remains unclear. In the present study, we have performed a weighted gene co-expression network analysis (WCGNA) to investigate links between INO80 expression and breast cancer sub-classification and progression. Our analysis revealed that INO80 repression is associated with differential responsiveness of estrogen receptors (ERs) depending upon breast cancer subtype, ER networks, and increased risk of breast carcinogenesis. To determine whether INO80 loss induces breast tumors, a conditional INO80-knockout (INO80 cKO) mouse model was generated using the Cre-loxP system. Phenotypic characterization revealed that INO80 cKO led to reduced branching and length of the mammary ducts at all stages. However, the INO80 cKO mouse model had unaltered lumen morphology and failed to spontaneously induce tumorigenesis in mammary gland tissue. Therefore, our study suggests that the aberrant function of INO80 is potentially associated with breast cancer by modulating gene expression. INO80 mutation alone is insufficient for breast tumorigenesis.

## 1 Introduction

Breast cancer is a devastating disease caused by genetic and epigenetic aberrations, which lead to alterations in gene expression and subsequently cellular functions. Numerous genetic mutations in epigenetic factors have been identified so far, and may play a key role in breast cancer development and therapy resistance (Wang et al., 2007; Cancer Genome Atlas Network, 2012; Stephens et al., 2012; Wang et al., 2014b; Helming et al., 2014; Kumar et al., 2016; Chu et al., 2017; Nickerson et al., 2017; Swinstead et al., 2018; Li et al., 2021). Among such epigenetic factors, ATP-dependent chromatin remodelers have emerged as potential biomarkers for breast cancer due to their role in regulating a distinct set of gene expression programs. For example, approximately 11% of breast cancer is related to mutations of the SWI/SNF complex, a member of the ATP-dependent chromatin remodeler family, and their roles are implicated in breast cancer cell plasticity and therapeutic response (Kadoch et al., 2013; Helming et al., 2014; Hohmann and Vakoc, 2014; Chu et al., 2017; Nagarajan et al., 2020; Xu et al., 2020). Mechanistically, dysregulation of such chromatin remodelers in breast cancer alter DNA compaction and accessibility, resulting in changes in 3D epigenomic and transcriptional profiles, particularly leading to aberrant expression of oncogenes (Bochar et al., 2000; Guerrero-Martínez and Reyes, 2018; Nagarajan et al., 2020; Kim et al., 2021). It is still unclear, however, whether the ATP-dependent chromatin remodelers are drivers or mere passengers of tumorigenesis as the factors are also linked to mammary stem cell function and mammary gland development in mice (Smalley and Ashworth, 2003; Cohet et al., 2010; Devinoy and Rijnkels, 2010; Macias and Hinck, 2012; Dravis et al., 2018; Holliday et al., 2018; Hanin and Ferguson-Smith, 2020; Ivanova et al., 2021).

Recent studies have shown that both fetal and adult basal cells share common epigenetic features and multi-lineage differentiation potential, and that the transcription factor SOX10 is critical for lineage determination of mammary epithelial cells and breast cancer metastasis (Dravis et al., 2015; Dravis et al., 2018). These findings suggest that breast cancer cells often acquire epigenetic and transcriptional features similar to those of the developing mammary gland. Understanding these similarities may provide insights into the underlying mechanisms of breast cancer development and help identify potential therapeutic targets. Studies have shown that the ATP-dependent chromatin remodeler families are essential for luminal cell identity and promote cell cycle decisions during mammary gland development (Cohet et al., 2010; Serber et al., 2012; Skibinski et al., 2014; Frey et al., 2017). Depletion of these factors results in attenuation of multiple signaling pathways crucial to regulation of mammary epithelial cell fate decisions and proliferation (Skibinski et al., 2014; Frey et al., 2017).

INO80 complex, a member of the ATP-dependent chromatin remodeler family, is involved in multiple functions related to cancer stem cells and cancer progression, through both canonical and non-canonical INO80 complexes that directly modulate chromatin architecture and gene expression (Min et al., 2013; Wang et al., 2014a; Lafon et al., 2015; Runge et al., 2018). Aberrant INO80 function has been associated with progression of multiple types of cancer through its binding to key enhancer and super-enhancer elements involved in oncogenic gene expression, including CXCL5 and MAP3K1 in non-small-cell lung cancer (Zhang et al., 2017), BMPR1A in live cancer (Wang et al., 2019), and MITF and SOX9 in melanoma (Zhou et al., 2016a). Furthermore, silencing of INO80 appears to have a similar effect to dysfunction of KRAS, MYC, PIK3CA, and ERRB2, inhibiting the migration and metastatic abilities of cancer cells (Zhang et al., 2017). While the association between INO80 and breast cancer has been demonstrated elsewhere (Segala et al., 2016), the specific function of the INO80 subunit and the underlying molecular mechanism involved in mammary development and breast cancer have not been fully elucidated.

In this study, we aimed to explore the functional role of INO80 in both breast cancer progression and mammary gland development through analysis of publicly available TCGA datasets and a conditional knockout (cKO) mouse model. Our findings demonstrate a significant, albeit heterogeneous, correlation between INO80 expression and breast cancer progression. Additionally, our study provides evidence to suggest that INO80 loss alone is not sufficient to induce the development of breast cancer in mice.

## 2 Materials and methods

### 2.1 Mouse models

All animal experiments in the present study were performed under the guidelines of the Institute of Animal Care and Use Committee of Konkuk University (IACUC# KU21020). The Ino80 cKO allele (Ino80^2f/2f^) and Tg(MMTV-Cre) animals were obtained from the Institut Clinique de la Souris (ICS; llkirch, France) and the Jackson laboratory. Female Ino80^2f/2f^ mice were bred with Ino80^2f/+^; MMTV-Cre males to produce littermate control (Ino80^2f/2f^) and experimental (Ino80^2f/2f^; MMTV-Cre) females. PCR genotyping for the Ino80 cKO allele was carried out with the following primers: 5′-AGG​CCT​TAT​TTA​GCT​CAG​GTT​GGC-3’ (forward) and 5′- CCA​CTA​CAC​ACA​GCA​GAT​ACA​CAT -3’ (reverse). The PCR amplicons for wildtype and the conditional alleles were 224 and 382 bp, respectively.

### 2.2 Tissue collection and whole-mount carmine staining

Mammary gland samples were harvested 4, 8, and 16 weeks after birth. Samples from inguinal mammary glands (#1, #2, and #5) on the right side were quickly frozen in liquid nitrogen (LN2) and then kept at −80°C, allowing RNA and protein extraction at later stages. The inguinal mammary glands (#2 and #4) were harvested and fixed in 4% paraformaldehyde (PFA)/PBS overnight at 4°C. After fixation, the glands were preserved in 70% EtOH/PBS for long-term use if needed. The procedure for whole-mount carmine staining was as described in a previous study (Seong et al., 2018). Briefly, samples were fixed in 4% PFA/PBS for 4–6 h, and then lipids were removed from the fat pad with Clarke’s solution (25% acetic anhydride in 75% EtOH) for 16–18 h at room temperature (RT). The slides were washed with 70% EtOH/PBS and PBS, followed by staining in Carmine Alum (C1022; Sigma-Aldrich, Burlington, MA, United States) solution overnight at RT. Samples were rinsed with PBS and 70% EtOH/PBS before incubation in a de-staining solution (2% HCl in 70% EtOH/PBS) for 3–6 h. Tissues were dehydrated in a series of EtOH (70%, 95%, and 100%) and xylene for at least 3 h at each step and mounted on a cover glass. Images were taken using an Olympus SZX7 microscope (Olympus, Tokyo, Japan).

### 2.3 H&E staining and immunofluorescence

The histology samples were cut into small pieces, placed into cassettes, and submerged in 4% PFA/PBS overnight at 4°C. Then, the samples were dehydrated with 70%, 95%, 100%, and 100% EtOH for 1 h at each step, incubated in xylene for 4 times for 30 min each time, and finally twice in paraffin for 1 h each time. The paraffin blocks were cut to a 5 μm thickness. For H&E staining, sections were rehydrated and incubated in hematoxylin (#1051750500; Sigma-Aldrich) for 5 min, washed in distilled water before being placed in eosin, and then soaked in EtOH 95% for washing. In addition, slides were dried and washed with an EtOH-xylene mixture before mounting with the mounting solution (#25608-33-7; Sigma-Aldrich). For immunofluorescence (IF) staining, tissue samples were steamed in antigen retrieval buffer (#E-IR-R104; Elabscience, Houston, TX, United States) for 40 min to recover antigen epitopes. Non-specific binding was blocked with blocking solution (2% donkey serum plus 3% BSA and 0.1% Triton-X 100 in PBS) for 1 h at RT in advance. Sections were incubated with primary antibodies [anti-KRT18 (1:250, #ab133263; Abcam, Cambridge, United Kingdom), anti-SMA (1:250, #ab124964; Abcam), anti-Ino80 (1:200, #18810-1-AP; Proteintech, Chicago, IL, United States)] overnight at 4°C, incubated with secondary antibodies [anti-rabbit Alexa Fluor 568 (1:250, #ab175471; Abcam) and anti-mouse Alexa Fluor 488 (1:250, #ab150077; Abcam)] in the dark for 1 h, washed with 1X PBS thrice, and briefly counter-stained with DAPI (1:1000, #ab228549; Abcam). The sections were mounted and imaged using a confocal microscope (LSM800; Carl Zeiss, Oberkochen, Germany).

### 2.4 TCGA and METABRIC analysis

The analysis of breast cancer patient survival was conducted using the Kaplan-Meier Plotter (https://kmplot.com/analysis/index.php?p=service), as described in Nagy et al. (2018). In this analysis, the expression levels of INO80 were utilized to investigate their relevance to patient survival. The mean expression value of all INO80 probes was calculated, and patients were categorized based on the auto cut-off of INO80 expression, with default parameters applied for the analysis, including the ER subtype, overall survival (OS), distant metastasis-free survival (DMFS), and recurrence-free survival (RFS).

Data on invasive breast carcinoma (BRCA) legacy level 3 was obtained from The Cancer Genome Atlas (TCGA) cohort, and raw RNA-seq counts and clinical data (1108 primary tumors and 114 normal samples) were retrieved using the TCGAbiolinks R package. The data was filtered and normalized using the TCGAanalyze_Normalization function to adjust the GC-content effect on read counts. Normalized transcriptomics and clinical data of METABRIC dataset (1966 tumor and 133 normal samples) were retrieved from MetaGxData package and cBioportal, respectively. Patients with INO80 expression were classified into molecular subtypes, such as normal, basal-like, HER2-enriched, luminal A, luminal B, and normal-like, and by tumor stage in each database. Samples without standards were excluded from the analysis. The INO80 expression sample also groups based on the ER_IHC, PR_IHC, or HER2_IHC subgroups. After statistical analysis with unpaired t-tests, plots were generated using the ggpubr and ggplot2 packages to visualize the relative INO80 expression between breast cancer subtypes, IHC groups, and cancer stage classification.

### 2.5 Weighted gene co-expression correlation network analysis (WGCNA)

Clinical features, gene copy number variation (CNV) data, and gene expression profiles of patients with breast cancer were obtained from TCGA and METABRIC, and analyzed using cBioportal. Only patients with estrogen immunohistochemistry (IHC) staining and gene expression data were included in the analysis. Weighted gene co-expression network analysis (WGCNA, ver.1.70) was performed on mRNA data to identify modules of co-expressed genes based on GISTIC and IHC traits. Poorly expressed genes and outliers were removed, and data was normalized using the limma package, with the top 5,000 genes selected. A *β* = 4 threshold power was chosen based on co-expression similarity to the scale-free topology fit index curve (h = 0.9), and the tree cut parameters were set at 0.15 with a minimum module size of 30 genes. The highest correlated module was selected for downstream analysis. Specific gene networks were identified using unsupervised clustering, and heatmaps were generated using the pheatmap package (ver. 1.0.12). Gene ontology analysis was performed using DAVID (ver. 6.8) (Huang et al., 2009a; Huang et al., 2009b), and gene–trait correlations were illustrated using gene significance (GS) scores and VisANT network analysis (Hu et al., 2004).

### 2.6 Quantification and image analyses

ImageJ (ver. 1.52, https://imagej.nih.gov/ij/) was utilized for the analyses of whole-mount carmine staining, immunofluorescence, and H&E staining in our study. The software enabled the measurement of various parameters, including the length of the mammary gland ductal tree in millimeters from the nipple to the last branch, the number of branching points, and the relative area of the fat pad in the mammary gland.

### 2.7 Statistical analyses

SigmaPlot (ver. 14; Systat Software, Chicago, IL, United States) and GraphPad Prism (ver. 5; GraphPad Software, La Jolla, CA, United States) were used for statistical analysis and producing graphs. Data are presented as mean and standard error of the mean (±SEM). Student’s t-test and one-way ANOVA with Bonferroni *post hoc* tests were used to determine statistical significance.

## 3 Results

### 3.1 INO80 expression in breast cancer

To investigate the contribution of the INO80 complex to breast cancer, we first examined the frequency of alteration in the expression of INO80 complex subunits in various cancer types using the cBioportal platform. To that end, a large amount of sequencing data from patients with cancer was retrieved from TCGA and METABRIC to investigate the impact of INO80 on breast cancer progression and outcome. As shown in Supplementary Figure S1A, INO80 complex subunits exhibit a high frequency of alteration in multiple cancer types. For instance, alterations in the INO80 complex were found in approximately 80% of non-small-cell lung carcinomas (PanCancer Atlas), and in approximately 60% of breast, lung, and colorectal cancers (PanCancer Atlas). Interestingly, INO80 showed an alteration frequency of 1%–8% in most cancer types and approximately 5% in all patients with breast cancer (Supplementary Figure S1B).

**FIGURE 1:**
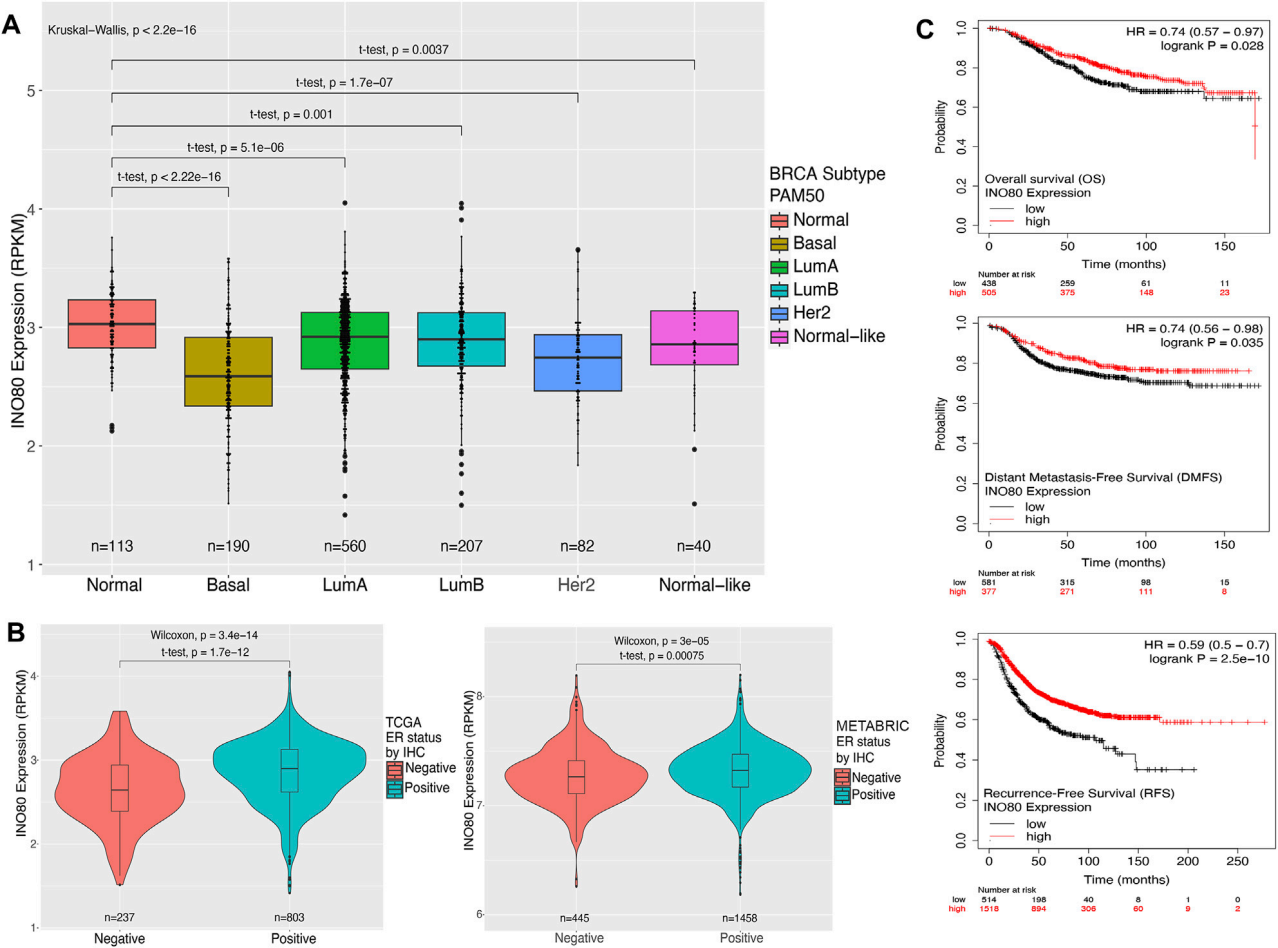
Expression and survival analysis of INO80 in breast cancer. **(A)** The level of INO80 expression in normal tissue and breast cancer subtypes. RNA-seq FPKMs values were retrieved from TCGA and analyzed based on clinical classification. Statistical analysis was performed using unequal t-tests, with significance set at *p* ≤ 0.05. **(B)** Comparison of INO80 expression between ER-positive and ER-negative cohorts in TCGA (left) and METABRIC data (right). Two cohorts were classified by immunohistochemistry (IHC) clinical data on ER and INO80 FPKMs between groups and statistically analyzed using the *t*-test with unequal sample sizes. **(C)** Kaplan–Meier survival analysis of patients with breast cancer and INO80 mutation and wild-type (WT) groups was conducted using the DriverDBv3 tool. The analysis used default parameters with gene symbols and means on the survival function, with overall survival (OS—top), disease-specific survival (DSS—center), and progression-free survival (PFI—bottom). BRCA, Breast Cancer LumA; Luminal subtype A; LumB, Luminal subtype B; HER2, Human epidermal growth factor receptor 2 subtype; PAM50, Prediction Analysis of Microarray 50; RPKM, reads per kilobase of transcript per million reads mapped.

Next, we aimed to determine the expression levels of INO80 in normal tissue and different breast cancer PAM50 subtypes, including basal, luminal A, luminal B, HER2, and normal-like. The PAM50 approach is closely associated with pathological classification and provides a means to identify or characterize cancer subtypes using RNA analysis rather than traditional histological staining methods. PAM50 intrinsic breast cancer subtypes, along with the associated proliferation score and risk of recurrence score (ROR-PT), are independent prognostic factors that enhance the classification of breast cancer patients into prognostic groups (Nielsen et al., 2010; Liu et al., 2016; Ohnstad et al., 2017). A remarkably lower expression of INO80 was found in all breast cancer subtypes, with significantly downregulated INO80 in the basal type compared with that in the normal sample (Figure 1A; Supplementary Figure S1C). Meanwhile, a relatively higher level of mean INO80 expression was found in patients with luminal breast cancer classified as ER and/or progesterone receptor (PR) IHC-positive (Figures 1A, B; Supplementary Figures S1C, D). Furthermore, there was no significant correlation between INO80 expression and tumor stage or HER2 IHC classification (Supplementary Figures S1E, F). On the other hand, lower expression of INO80 affects overall survival (OS) rate, Distant Metastasis-free survival (DMFS), and Recurrence-free survival (RFS) endpoints in breast cancer. The Kaplan-Meier survival analysis of the TCGA dataset revealed that breast cancer patients with lower INO80 expression had reduced survival probabilities over a 150-month follow-up period (Figure 1C).

### 3.2 INO80 mutations are commonly detected in human breast cancer and correlated with breast cancer subtype

**FIGURE 2:**
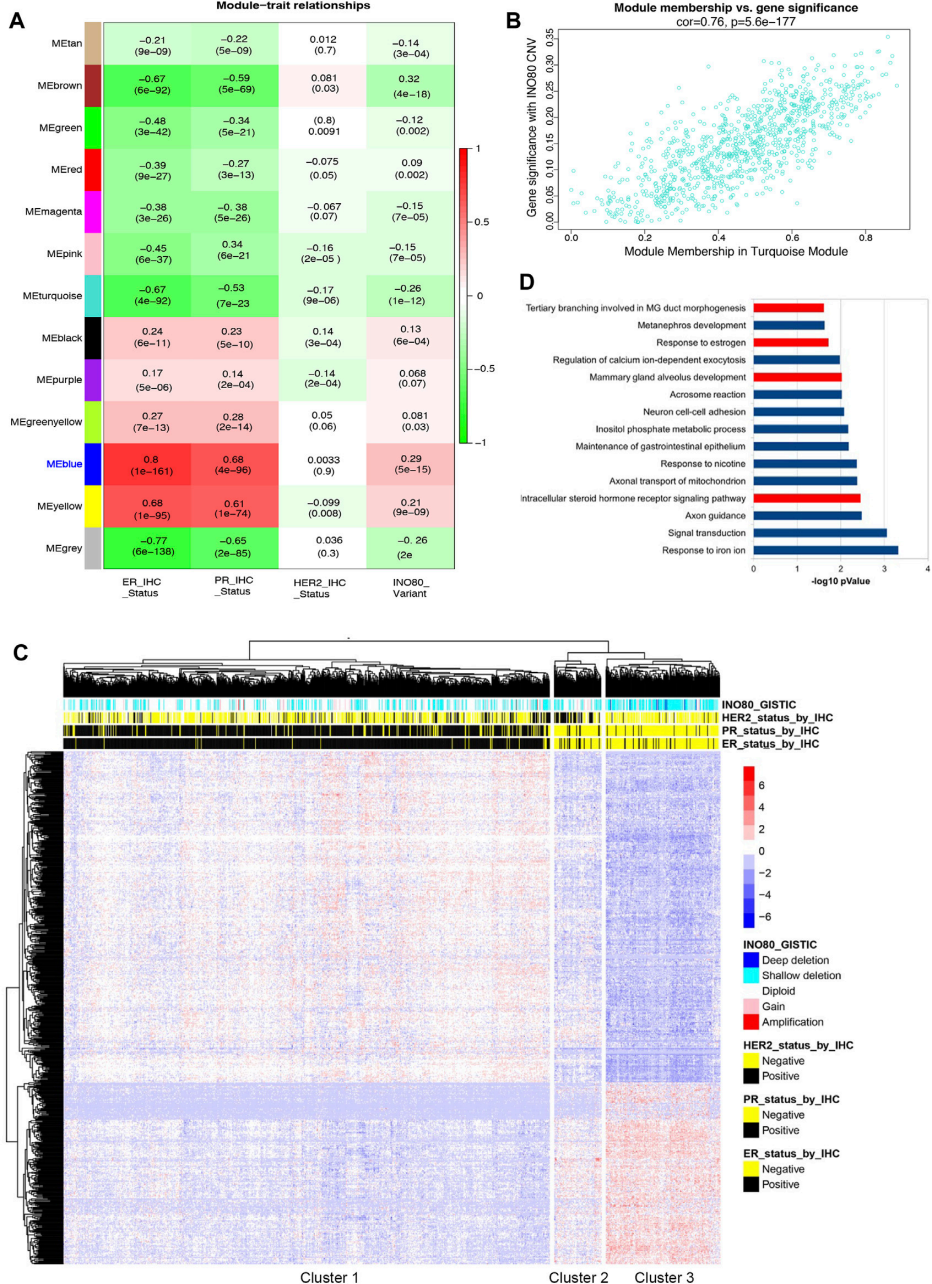
The correlation between INO80 expression level and breast cancer. **(A)** Weighted gene co-expression network analysis (WGCNA) was performed to identify the correlation between INO80 copy number variation (CNV) and gene set modules. The MEblue module was found most associated with INO80 CNV Genomic Identification of Significant Targets in Cancer (GISTIC) among the 13 modules, where red indicates co-expression and green indicates negative co-regulation. **(B)** The correlation between INO80 copy number variation (CNV) and the significant module, MEblue (793 genes, correlation value 0.76, *p* = 5.6e-177). **(C)** A heatmap showing unsupervised clustering of subset genes in the MEblue module of TCGA. The relation between INO80 traits (IHC and CNV) and expression (z-score) of genes is classified into three clusters, with each cluster associated with a certain subtype of breast cancer. **(D)** The top 15 gene ontology biological processes (GOBPs) from DAVID analysis of gene set data overlapped genes between the MEblue module of TCGA and the MEblue module of METABRIC. GOBPs related to mammary gland development are highlighted in red. ER, estrogen receptor; PR, progesterone receptor; MG, mammary gland; HER2, Human epidermal growth factor receptor 2; CNV, Copy Number Variation.

Given the significant increase in the risk of breast cancer and decreased survival rate observed in patients with INO80 complex dysfunction (Figure 1C), as well as the need for further investigation into the association between INO80 deregulation and breast cancer, WGCNA was performed to investigate gene expression, CNV, and clinical IHC annotations of INO80 in patients with breast cancer (Cancer Genome Atlas Network, 2012; Ciriello et al., 2015) followed the workflow in Supplementary Figure S2A. Our analysis primarily focused on the effect of INO80 mutation on breast cancer subtype and changes in gene cohorts associated with breast cancer. The gene expression profile was divided into 13 co-expression modules, each containing 34–687 genes (Figure 2A). Notably, the MEblue module was strongly correlated with INO80 CNV (cor = 0.76), with a significant *p*-value of 1.3e-128 (Figure 2B). This cohort contained a subset of genes that could be considered potential biomarkers for breast cancer progression, including FOXA1, MLPH, ESR1, AR, GATA3, TFF1, THSD4, and TBC1D9 (Supplementary Figure S2B). Moreover, analysis of the ER-associated co-expressed network uncovered several key genes involved in ER signaling, such as ESR1, AR, GATA3, and TFF1, and showed a strong correlation between differential gene expression in the MEblue cohort and INO80 CNV. Based on gene expression and clinical patient data, our analysis classified three clusters and showed that INO80 deletion, including both shallow and deep deletion, was associated with IHC status (Figures 2A, C).

Based on INO80 CNV and IHC status, the unsupervised clustering identified three clusters: the primary luminal (cluster 1), mainly composed of patients with ER+/PR+/HER2-status; the primary HER2+ (cluster 2), mainly composed of patients with ER-/PR-/HER2+ status; and the primary triple-negative breast cancer (TNBC) (cluster 3), mainly composed of patients with ER-/PR-/HER2-status (Figure 2C). The odds ratio (OR) suggests that the risk of breast cancer is 6.5724 times higher in patients with INO80 deletion in the TNBC cluster than in those with other mutations, which is statistically significant [CI (4.55–9.49) and *p* < 0.0001]. Similarly, the OR shows that the risk of breast cancer is 5.1429 times higher in cluster 3 than in cluster 2 (HER2+ subtype) [CI (2.75–9.63) and *p* < 0.0001]. Additionally, the analysis of the METABRIC dataset showed consistency with our TCGA data analysis, demonstrating a significant correlation between INO80 CNV and luminal signatures, as well as an association with the TNBC cluster compared to other breast cancer subtypes (Supplementary Figures S2D, E). The OR for the primary TNBC cluster was 4.1385 times higher than that for the primary HER2 cluster [CI (2.8158–6.0825), *p* < 0.0001] and 7.1482 times higher than that for the primary luminal cluster [CI (5.3870–9.4853), *p* < 0.0001]. Therefore, loss of INO80 likely increases the risk of breast carcinogenesis, especially in the TNBC subtype.

Up to 189 genes overlapped between 2 cohorts (MEblue from TCGA analysis and MEblue from METABRIC analysis), accounting for 25%–30% of the total genes in each cohort (Supplementary Figure S2F). Gene ontology (GO) term analysis showed that the MEblue genes were associated with mammary gland epithelial cell differentiation and mammary gland duct branching morphogenesis (Supplementary Figure S2G). The top list of gene ontology biological processes (GOBP) was obtained by overlapping two modules associated with INO80 CNV, including terms related to mammary gland development and estrogen regulation (Figure 2D). Therefore, the findings suggest an underlying mechanism by which INO80 function is involved in mammary gland development and breast cancer.

### 3.3 INO80 mutation impairs mammary gland development

**FIGURE 3:**
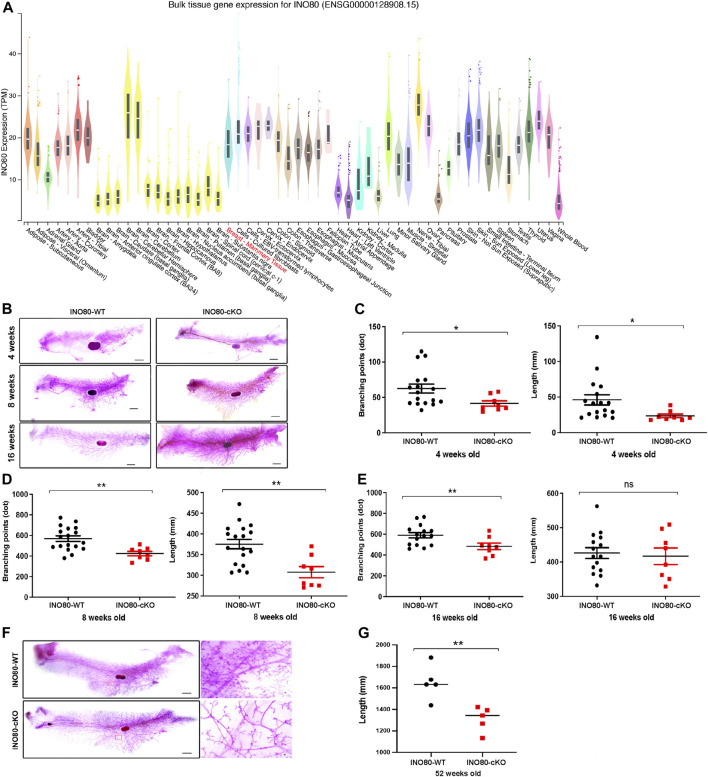
INO80 loss impairs mouse mammary gland development. **(A)** INO80 expression in bulk RNA sequencing of GTEx data. **(B)** Whole-mount carmine staining of mammary glands from 4-, 8-, and 16-week-old mice. **(C–E)** Quantification of branching points and length of mammary glands from INO80 cKO mice versus those from wild-type mice at 4-weeks old [in **(C)**], 8-weeks old [in **(D)**], and 16 weeks old [in **(E)**]. **(F,G)** The mammary gland of 52-week-old mice stained with whole carmine [in **(F)**] and the length quantification of mammary gland ductal analysis with 5 mice per group. Ductal length was significantly decreased in INO80-cKO compared with that in WT mice, which was consistent with the data on development stages [in **(B)**]. Significant statistic: *p-value =< 0.05, **p-value =< 0.005; GTEx, Genotype-Tissue Expression; WT, wide type; cKO, Conditional knockout.

INO80 is highly expressed in the lungs, colon, breasts, and several parts of the brain, such as the nerves, cerebellar hemisphere, and cerebellum (Figure 3A). To investigate whether INO80 plays a role in tumorigenesis, a mouse model was generated in which the INO80 gene was explicitly deleted in the mammary gland. Complete elimination of the INO80 gene in mice results in embryonic lethality (Min et al., 2013; Wang et al., 2014a; Qiu et al., 2016); therefore, we utilized a Cre-loxP system with Tg(MMTV-Cre) mice (line D) (Wagner et al., 1997; Wagner et al., 2001) to generate control (INO80^f/f^) and cKO [Tg(MMTV-Cre); INO80^f/f^] females (Supplementary Figure S3A). Genotyping analysis confirmed that the INO80 cKO mice were viable (Supplementary Figure S3B), and their live offspring displayed an expected Mendelian ratio (Table 1). Immunofluorescence confirmed suppression of the INO80 expression in the ductal lumen of the knockout mouse model (Supplementary Figure S3C).

**TABLE 1:** Mouse (female only) genotyping result.

Parent	INO80^2f/-^;MMTV-Cre (♂) X INO80^2f/2f^ (♀)			
	INO80^2f/2f^ or INO80^2f/+^	INO80^2f/+^;MMTV-Cre	INO80^2f/2f^;MMTV-Cre	Total
Observation	101 (48.6%)	60 (28.8%)	47 (22.6%)	208
Expectation	104 (50%)	52 (25%)	52 (25%)	208

Chi-square: 1.798, Degrees of freedom: 2, *p*-value: 0.40696078, Yates’ chi-square: 1.531, Yates’ *p*-value: 0.46510132. By conventional criteria, this difference is considered to be not statistically significant.

Next, to determine whether the loss of INO80 causes phenotypic changes in mammary tissue, whole-mount carmine staining was performed on mammary glands harvested from 4-week-, 8-week-, and 16-week-old INO80 cKO and littermate control females (Figure 3B). The staining was used to examine the ductal growth within the mammary glands in juvenile and adult virgin mice. Quantification analysis of the whole-mount carmine staining revealed that the number of measurable branching points was reduced, and mammary duct length was significantly decreased in the INO80 cKO mice compared to values in control mice (Figures 3C–E). The data suggest that INO80 deficiency resulted in hypoplasia in developing mouse mammary glands. Next, to investigate whether the loss of INO80 is sufficient to induce mammary tumorigenesis, we examined the lifespan and mammary gland morphology of cKO mice up to 2 years of age. Our analysis showed no incidence of spontaneous mammary tumorigenesis in the INO80 cKO mice. Although there were no significant lifespan differences between the INO80 cKO and control mice for up to 2 years, morphometric analysis showed consistent reductions in the length and branching points of mammary glands in the INO80 cKO mice (Figures 3F, G). Notably, histological examination of the mammary glands using CK18(+)/α-SMA(+) ratio and H&E staining revealed the presence of morphologically normal-like ductal structures in the INO80 cKO mammary glands (Supplementary Figures S3D, E). Furthermore, the degree of mammary gland hypoplasia was alleviated in pregnant and lactating INO80 cKO mice (Supplementary Figure S3E), which may account for the comparable weight of newborn pups between the two groups (Supplementary Figure S3F). Therefore, our findings suggest that INO80 loss alone is insufficient to induce mammary tumor formation in mice.

## 4 Discussion

Understanding the intricate molecular mechanisms underlying normal mammary gland development is crucial for developing effective treatment strategies for breast cancer, which is the most prevalent cancer in women worldwide, accounting for 24.5% of all cancer cases, and is the fifth leading cause of cancer-related death among women, responsible for 15% of all cancer deaths (Sung et al., 2021; Siegel et al., 2022). The mammary gland undergoes numerous phases of development and differentiation, including ductal tree expansion and invasion into the fat pad, pregnancy, lactation, and involution stages (Macias and Hinck, 2012; Biswas et al., 2022), all of which are tightly regulated by a complex interplay between genetic and epigenetic factors (Bae and Hennighausen, 2014; Holliday et al., 2018).

Driver epi-mutations can disrupt normal mammary development and promote breast cancer progression, while passenger epi-mutations are typically neutral and do not provide a growth advantage to cancer cells, but may be associated with functionally altered signaling pathways (McFarland et al., 2017; Fernandez-Moya et al., 2020; Ying and Beronja, 2020). For instance, the absence of certain ATP-dependent chromatin remodeling factors of the SWI/SNF complex affects mammary gland development (Cohet et al., 2010; Skibinski et al., 2014), which has helped clarify the specific roles of these factors in breast cancer development and resistance to therapy (García-Pedrero et al., 2006; Nagarajan et al., 2020; Xu et al., 2020).

INO80 is essential for cell reprogramming, blastocyst development (Wang et al., 2014a; Zhou et al., 2016b), and organ development, including in spermatogenesis (Serber et al., 2016; Chakraborty and Magnuson, 2022), ventricular compaction, and coronary vascularization during heart development (Rhee et al., 2018, 2021). Recent studies have shown that complete abolition of INO80 causes embryonic lethality in mice (Min et al., 2013; Wang et al., 2014a; Lee et al., 2014; Lafon et al., 2015; Qiu et al., 2016) by altering compaction, accessibility of DNA within chromatin, and various molecular processes, including DNA replication, transcription, and DNA damage response (Shen et al., 2000; Gospodinov et al., 2011; Lange et al., 2011; Volokh et al., 2016). Furthermore, this complex has been implicated in both maintenance of stem cell and progression of cancer cell by functioning as a critical regulator of super-enhancers in both contexts (Wang et al., 2014a; Zhou et al., 2016a; Serber et al., 2016; Zhang et al., 2017). Additionally, INO80 is required for H2A.Z dynamics in ER signaling, and silencing of INO80 reduces stimulation of endogenous GREB1 and TFF1 enhancers in breast cancer (García-Pedrero et al., 2006; Papamichos-Chronakis et al., 2011; Segala et al., 2016). Therefore, these findings, along with our INO80 cKO study, suggest that INO80 plays a key role in mammary gland development and breast cancer progression.

The INO80 complex comprises highly conserved principal subunits in humans, mice, flies, and yeast (Shen et al., 2000, 2003; Jin et al., 2005). The canonical INO80 complex comprises several subunits, including INO80, RUVBL1, RUVBL2, MCRS1, and YY1, and is involved in active transcription regulation by physically interacting with P300 and MED1. This complex is also associated with active histone modifications, including H3K4me1, H3K4me3, and H3K27ac (Zhou et al., 2016a; Zhang et al., 2017; Runge et al., 2018). RUVBL1 and YY1 promote tumor growth (Wang et al., 2015; Fan et al., 2017), and inhibiting RUVBL1 expression in metastatic breast cancer cells can reduce both cell proliferation and invasion (Fan et al., 2017). Additionally, YY1 promotes tumor growth by suppressing the expression of p27 and interacting with it (Wang et al., 2015). Conversely, the noncanonical class of the INO80 complex is linked to a repressive histone modification, H3K27me3, suggesting that the INO80 complex can act as a tumor suppressor (Runge et al., 2018; Chakraborty and Magnuson, 2022). INO80 occupancy affects replication forks, and its silencing can activate the replication stress-induced ATR-CHK1 signaling pathway in colon cancer (Lee et al., 2017).

In breast cancer, INO80 expression is generally lower, although it correlates with the ER-positive breast cancer subtype (Figures 1A, B). Higher median INO80 expression was found in ER-positive than in ER-negative breast cancer. Furthermore, the results from our WGCNA analysis indicated the existence of unknown networks between INO80 and a subset of luminal breast cancer biomarkers, including FOXA1, ESR1, GATA3, TFF1, and AR (Supplementary Figure S2B). The FOXA1 transcription factor is a key regulator of breast cancer identity, as it controls ER activity (Hurtado et al., 2011). Furthermore, FOXA1 and MLPH upregulate the expression of luminal-like genes, and both have emerged as prognostic indicators for breast cancer (Thorat et al., 2008; Hurtado et al., 2011; Bernardo et al., 2013; Thakkar et al., 2015). Some genes in ER-associated co-expressed networks alter the gene expression profile of luminal breast cancer cells and are predictive of patient response to breast cancer therapy (Tozlu et al., 2006; Chen et al., 2011; Zhu et al., 2020). THSD4 and TBC1D9 are two factors that drive TNBC, and their expression is also linked to the epithelial–mesenchymal transition (EMT), metastatic dissemination, and the plasticity of breast cancer cells (Cohen et al., 2014; Kothari et al., 2020; Miao et al., 2020; Kothari et al., 2021). Previous studies have also revealed the opposing effects of GATA3 function on the tumor suppressor gene THSD4 (Cohen et al., 2014). These findings suggest a potential role for INO80 in ER signaling, where the presence of INO80 is required for ER activity by forming a physical interaction with the ER-INO80 complex in breast cancer, as reported in previous studies (Segala et al., 2016). Moreover, our analysis suggests that there is an increasing risk of breast cancer and reduced survival rates in patients harboring the INO80 mutation. In addition, unsupervised clustering from the WGCNA analysis revealed an association between patients with INO80 mutation and breast cancer biomarkers (ER/PR/HER2 IHC data). INO80 copy number status is associated with breast cancer subtype, whereas patients with primary breast cancer and TNBC subtypes harbored higher odd ratios than did those with luminal or HER2 subtypes (Figure 2C; Supplementary Figure S2C).

The gene cohort from the WGCNA analysis suggests that INO80 may be involved in mammary gland development (Figure 2D; Supplementary Figure S2F), which is consistent with our *in vivo* model showing that INO80 affects ductal morphogenesis in the mammary gland (Figures 3C–E, G). Several factors, including steroid hormone, BMP, Wnt, cell cycle, and peptide hormone signaling pathways, are known to play roles in mammary gland development (Bocchinfuso et al., 2000; Feng et al., 2007; Hens et al., 2007; Timmermans-Sprang et al., 2019). Previous studies have demonstrated the involvement of INO80 in stem cell differentiation and mammary tumors via the Wnt pathway (Wang et al., 2014a; Zhou et al., 2016b; Zhang et al., 2017; Timmermans-Sprang et al., 2019) and its regulation of BMP signaling in embryonic and liver cancer stem cells (Hens et al., 2007; Qiu et al., 2016; Wang et al., 2019). Another study demonstrated that the overexpression of INO80 and NANOG could promote cervical cancer cell proliferation and tumorigenesis (Hu et al., 2016). It is possible that the downregulation of these signaling pathways by INO80 KO led to changes in the mammary gland phenotype in our model. INO80 loss in our mouse model failed to cause tumor formation (Figure 3F). We cannot rule out the possibility of insufficient or unexpected CRE activity in the Tg(MMTV-cre) line (De Vos et al., 2008; Diegel et al., 2010). However, based on our breast cancer data analysis and the observed mammary gland phenotype, it is reasonable to hypothesize that INO80 may play a role in enhancing signaling abnormalities associated with breast cancer oncogenesis. Nevertheless, further investigation is required to determine the exact nature of INO80’s involvement in breast cancer development and progression.

## Data Availability

The original contributions presented in the study are included in the article/Supplementary Material, further inquiries can be directed to the corresponding author.

## Supplementary material

SUPPLEMENTARY FIGURE S1 Expression of INO80 components in breast cancer. **(A)** Copy number variation analysis of subunits in the INO80 complex obtained from TCGA. The cBioportal tool was used for the analysis. Note that up to 60% of INO80 complex alterations are associated with breast cancer. **(B)** Histogram showing that the alteration frequencies of INO80 in Pan-Cancer Atlas patients differ between cancer types. The average INO80 alteration frequency in breast cancer datasets can be as much as 5%. **(C, D)** Violin plots showing INO80 FPKMs expression based on PR **(C)** and HER2 IHC clinical data **(D)** generated from TCGA (top) and METABRIC (bottom) datasets. A statistical significance between the groups was found only in the PR group. **(E)** Boxplot assessed the expression of INO80 in each breast cancer subtype using the MetaGxBreast R package. The statistics were analyzed using unpaired t-tests, comparing each breast cancer subtype with the corresponding normal subset. **(F)** Correlation between INO80 expression and tumor stage (I, II, III, and IV) in TCGA (left) and METABRIC (right) databases. An unpaired t-test was used for statistical analysis, with significance set at *p* ≤ 0.05. CNV, Copy Number Variation; CNA, Copy Number Alteration; IHC, immunohistochemistry; RPKM, reads per kilobase of transcript per million reads mapped.

SUPPLEMENTARY FIGURE S2 The correlation between INO80 and breast cancer. **(A)** The workflow of WGCNA analysis using TCGA and METABRIC breast cancer data. **(B)** Network of MEblue (TCGA) genes identified in the WGCNA analysis. Each node represents a gene, and lines show the known connections. **(C)** A correlation plot between INO80 CNV and genes of the MEblue (METABRIC) module. **(D)** Relationship between modules (subsets of genes) and INO80 traits (IHC status and CNV). Red indicates co-expression, and green presents no correlation in expression. **(E)** Unsupervised clustering of METABRIC data analyzed by the WGCNA. Heatmap showing the correlation between gene expression and INO80 traits (IHC status and CNV) of the MEblue module. **(F)** A venn diagram showing overlapping genes between the two most co-expressing modules based on INO80 CNV and METABRIC data. **(G)** Top 15 GOBPs in the MEblue module of the WGCNA analysis using TCGA data. ER, estrogen receptor; PR, progesterone receptor; MG, mammary gland; HER2, Human epidermal growth factor receptor 2; CNV, Copy Number Variation.

SUPPLEMENTARY FIGURE S3 Characterization of INO80 cKO mice. **(A)** A breeding scheme to produce the INO80 conditional knockout (cKO) mouse model. Exon 6 of the INO80 gene is flanked by loxP sites and deleted by the Tg(MMTV-Cre) line. **(B)** PCR results of offspring genotyping where WT (224 bp band), hemizygous (242 and 382 bp bands), and homozygous (382 bp band) of INO80 gene. **(C)** Confirmation of INO80 depletion by immunofluorescence with INO80 antibody (red), CK18 antibody (luminal cell—turquoise) or alpha-SMA antibody (basal cell—turquoise), and DAPI (blue). **(D)** GraphPad Prism illustrates the ratio between luminal cells and basal cells, and an unpaired t-test showed that the difference was insignificant. **(E)** H&E staining of postnatal (4, 8, and 16 weeks after delivery), pregnant (18 days post coitus), and lactating (lactation day 2, L2) mammary glands. **(F)** Measurement of body weight of pups delivered from WT or cKO females.
