# Supplementary figures and images for: INO80 function is required for mouse mammary gland development, but mutation alone may be insufficient for breast cancer
Source: Front Cell Dev Biol. 2023 Nov 1;11:1253274. doi: 10.3389/fcell.2023.1253274 (PMC10646318; doi:10.3389/fcell.2023.1253274)

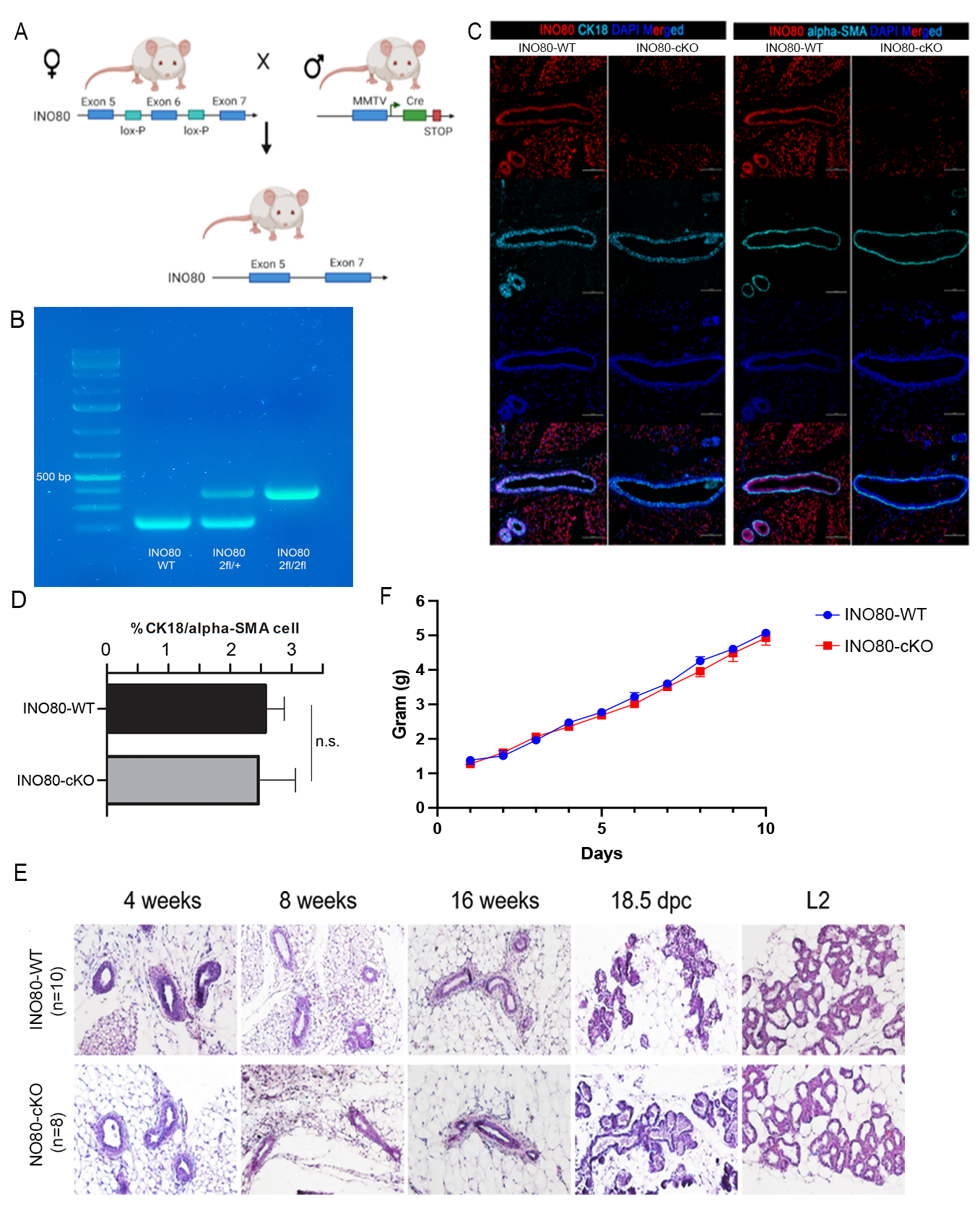

Supplement: Supplementary file 1 [file Image3.TIF]

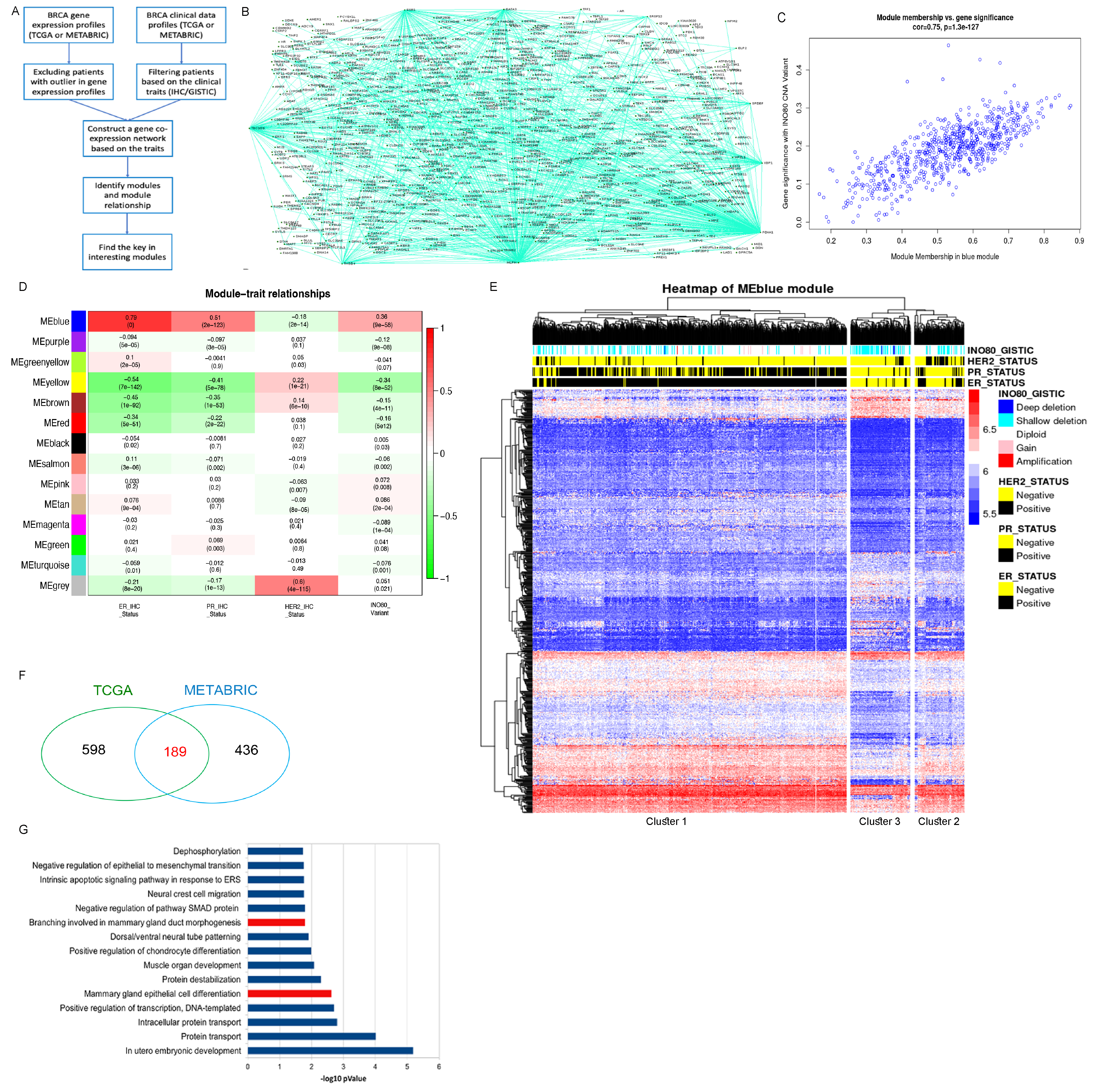

Supplement: Supplementary file 2 [file Image2.TIF]

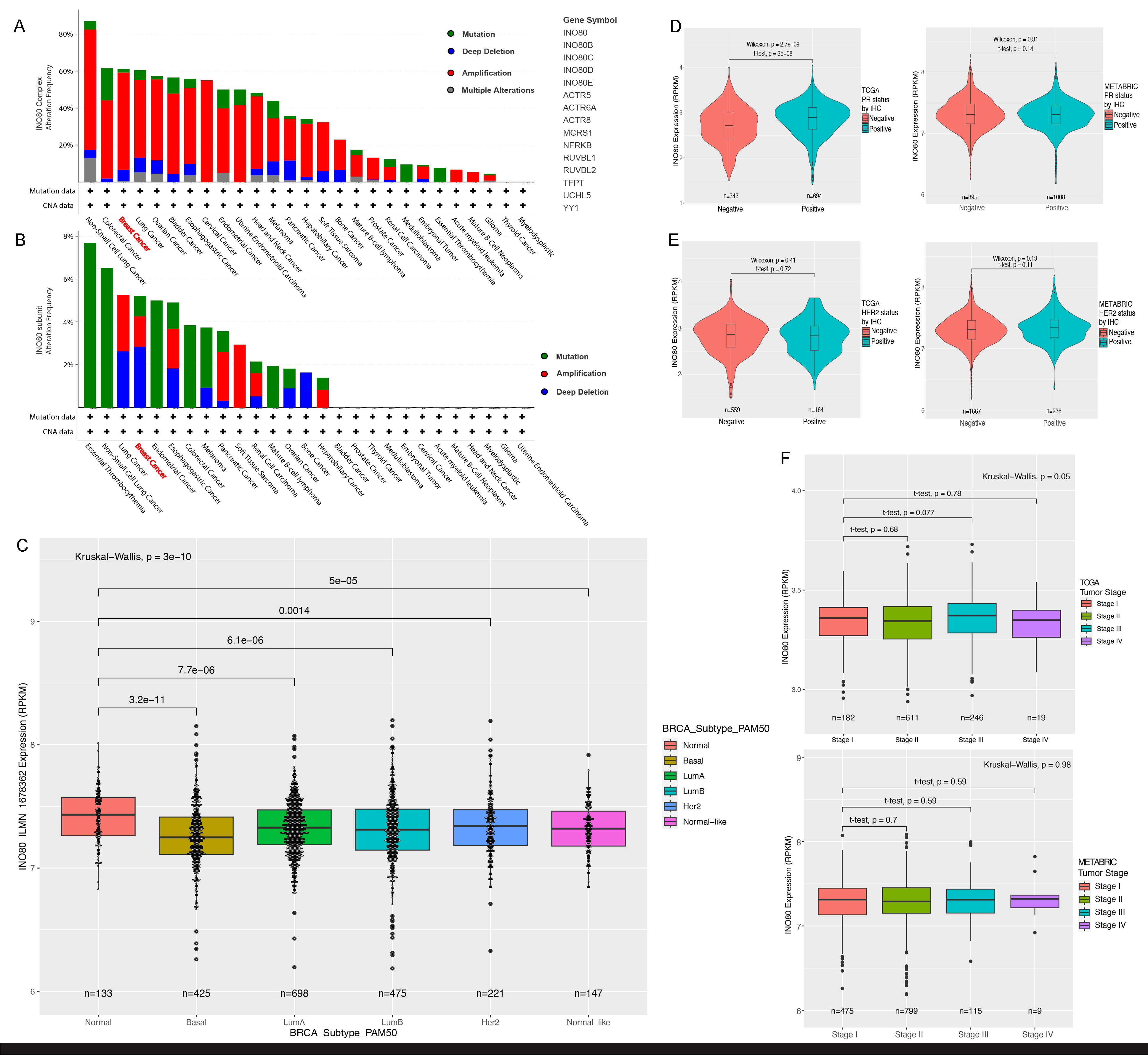

Supplement: Supplementary file 3 [file Image1.TIF]
